# The Use of Traditional, Complementary, and Integrative Medicine in Cancer: Data-Mining Study of 1 Million Web-Based Posts From Health Forums and Social Media Platforms

**DOI:** 10.2196/45408

**Published:** 2023-04-21

**Authors:** Chun Sing Lam, Keary Zhou, Herbert Ho-Fung Loong, Vincent Chi-Ho Chung, Chun-Kit Ngan, Yin Ting Cheung

**Affiliations:** 1 School of Pharmacy, Faculty of Medicine The Chinese University of Hong Kong Hong Kong China (Hong Kong); 2 Department of Clinical Oncology, Faculty of Medicine The Chinese University of Hong Kong Hong Kong China (Hong Kong); 3 School of Chinese Medicine, Faculty of Medicine The Chinese University of Hong Kong Hong Kong China (Hong Kong); 4 Jockey Club School of Public Health and Primary Care, Faculty of Medicine The Chinese University of Hong Kong Hong Kong China (Hong Kong); 5 Data Science Program, Worcester Polytechnic Institute Worcester, MA United States

**Keywords:** traditional, complementary, integrative, social media, cancer, forums, digital health, traditional, complementary, and integrative medicine, TCIM, perceptions, machine learning, cancer care

## Abstract

**Background:**

Patients with cancer are increasingly using forums and social media platforms to access health information and share their experiences, particularly in the use of traditional, complementary, and integrative medicine (TCIM). Despite the popularity of TCIM among patients with cancer, few related studies have used data from these web-based sources to explore the use of TCIM among patients with cancer.

**Objective:**

This study leveraged multiple forums and social media platforms to explore patients’ use, interest, and perception of TCIM for cancer care.

**Methods:**

Posts (in English) related to TCIM were collected from Facebook, Twitter, Reddit, and 16 health forums from inception until February 2022. Both manual assessments and natural language processing were performed. Descriptive analyses were performed to explore the most commonly discussed TCIM modalities for each symptom and cancer type. Sentiment analyses were performed to measure the polarity of each post or comment, and themes were identified from posts with positive and negative sentiments. TCIM modalities that are emerging or recommended in the guidelines were identified a priori. Exploratory topic-modeling analyses with latent Dirichlet allocation were conducted to investigate the patients’ perceptions of these modalities.

**Results:**

Among the 1,620,755 posts available, cancer-related symptoms, such as pain (10/10, 100% cancer types), anxiety and depression (9/10, 90%), and poor sleep (9/10, 90%), were commonly discussed. Cannabis was among the most frequently discussed TCIM modalities for pain in 7 (70%) out of 10 cancer types, as well as nausea and vomiting, loss of appetite, anxiety and depression, and poor sleep. A total of 7 positive and 7 negative themes were also identified. The positive themes included TCIM, making symptoms manageable, and reducing the need for medication and their side effects. The belief that TCIM and conventional treatments were not mutually exclusive and intolerance to conventional treatment may facilitate TCIM use. Conversely, TCIM was viewed as leading to patients’ refusal of conventional treatment or delays in diagnosis and treatment. Doctors’ ignorance regarding TCIM and the lack of information provided about TCIM may be barriers to its use. Exploratory analyses showed that TCIM recommendations were well discussed among patients; however, these modalities were also used for many other indications. Other notable topics included concerns about the legalization of cannabis, acupressure techniques, and positive experiences of meditation.

**Conclusions:**

Using machine learning techniques, social media and health forums provide a valuable resource for patient-generated data regarding the pattern of use and patients’ perceptions of TCIM. Such information will help clarify patients’ needs and concerns and provide directions for research on integrating TCIM into cancer care. Our results also suggest that effective communication about TCIM should be achieved and that doctors should be more open-minded to actively discuss TCIM use with their patients.

## Introduction

### Background

Despite recent advances in the diagnosis and treatment of cancer, patients and survivors of cancer treated with conventional cancer therapies often experience treatment-related toxicities, chronic symptoms, and poorer health-related quality of life [1]. Therefore, many patients with cancer undergo other approaches, including traditional, complementary, and integrative medicine (TCIM) [2]. TCIM refers to “a broad set of healthcare practices that are not part of conventional medicine and are not fully integrated into the dominant healthcare system” [3]. These include traditional medicines based on theories, beliefs, and experiences indigenous to different cultures (eg, traditional Chinese medicine and Ayurvedic medicine) and other complementary medicine modalities (eg, dietary supplements and homeopathy; Table S1 in Multimedia Appendix 1) [3,4]. Integrative medicine combines conventional and complementary approaches in a coordinated manner [4]. A recent review reported that approximately half of patients with cancer globally use TCIM modalities [3], such as yoga, meditation, homeopathy, and supplements [5,6].

The decision-making process by which patients with cancer decide to use TCIM is complex and dynamic. Patients continuously seek information about TCIM and validation from the experiences of others to fulfill different needs throughout their cancer journey, and they rely on a wide range of information sources [7]. Although patients with cancer prefer health advice from physicians, previous studies have found that 20% to 77% of patients do not disclose the use of TCIM to their physicians, and many oncologists do not actively discuss it with patients [8]. Although patients obtain limited information on TCIM from their oncologists, the internet remains one of the most common sources of information on TCIM [9]. Cancer-specific web-based communities are flourishing, enabling patients and caregivers to gain access to health information and share their perspectives with others [10]. Recent studies have leveraged such patient-generated data from social media platforms and web forums to gather the experiences and concerns of patients with cancer on contemporary treatments [11,12].

Despite the popularity of TCIM among patients with cancer, few related studies have used data from web forums and social media platforms to explore the use of TCIM among patients with cancer [13,14]. A French study of breast cancer forums and Facebook groups found that physical and nutritional interventions were commonly used by patients with breast cancer [13]. Another Italian study performed manual sentiment analyses on different TCIM modalities and found that “biologically based therapies or nutrition” and the Italian “Di Bella multitherapy” were the most commonly used modalities [14]. However, these studies were confined to local data and did not provide information about the patients’ interest in TCIM for treating their cancer and related symptoms. Moreover, they did not identify any concerns regarding TCIM use by patients. Manual content analysis of web-based forums requires substantial human effort and is time-consuming; thus, it is usually limited to analyzing a relatively small number of posts and revealing the occurrence of related terms [13,15,16]. In contrast, machine learning methods can help discover latent topics or patterns hidden within a large amount of unstructured text and can also predict the attitudes and opinions expressed in posts on social media [17]. Machine learning methods, such as latent Dirichlet allocation (LDA), have been used in some recent studies to analyze social media data to identify patients’ perceptions and concerns [18,19]. However, these methods have not been used to study the use of TCIM in patients with cancer and their perceptions that reflect their view of TCIM from their expectations and experiences of seeking information and using these modalities [20].

### Objective

Considering the emerging popularity of TCIM among patients with cancer, it is important for health care professionals to understand patients’ reasons and perceptions of TCIM use and have better communication with patients about TCIM use. Moreover, most studies on patients’ behaviors and perceptions concerning TCIM have been conducted in oncology centers or hospitals, which might differ from those of the web-based cancer community [2]. Therefore, we leveraged multiple health forums and social media platforms to explore the positive and negative perceptions of TCIM use by patients with cancer, as well as the facilitators and barriers to the use of TCIM. As TCIM encompasses a wide range of modalities, we also investigated the interest and receptivity of patients to different TCIM modalities for different cancer diagnoses and symptoms to inform future research in integrative oncology. Machine learning methods were used for exploratory analysis to reveal topics of interest and concerns related to the use of TCIM modalities that are either emerging or have been recommended in relevant guidelines and TCIM use for managing cancer-related symptoms.

## Methods

### Overview

**Figure 1:**
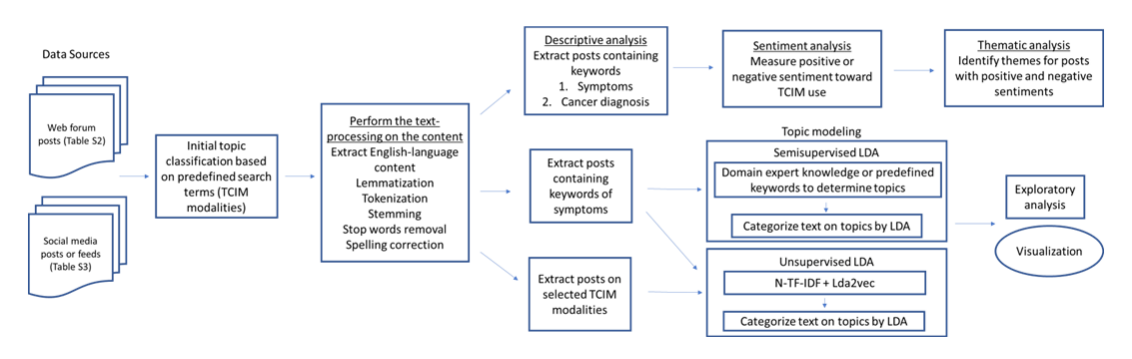
Workflow of analysis of posts from health forums and social media. Texts were preprocessed (lemmatization, tokenization, and stemming) to clean and prepare text data. Sentiment analysis was performed to score the sentiment of each post from −1 to 1 (a negative score refers to negative sentiment and a positive score refers to positive sentiment). For semisupervised latent Dirichlet allocation (LDA), traditional, complementary, and integrative medicine (TCIM) modalities were grouped according to the classification by the National Centre for Complementary and Integrative Health. Gensim LDA algorithms were used in both semisupervised and unsupervised LDA to categorize texts into different numbers of topics. Word clouds were generated from topic modeling to visualize the top keywords from each topic, and domain knowledge was used to explore themes related to each topic. TF-IDF: term frequency–inverse document frequency. Tables S2 and S3 are from Multimedia Appendix 1.

This retrospective study involved the collection and analysis of textual data from web forums and social media platforms. A combination of manual analysis and machine learning techniques (natural language processing [NLP]) was used for content and sentiment analyses. Content analysis refers to the objective and quantitative assessment of text characteristics, whereas sentiment analysis refers to the measurement of the polarity (positive, negative, and neutral) of the text [21,22]. A summary of the workflow, from data extraction to analysis, is presented in Figure 1.

### Data Sources

To increase the generalizability and power of the analyses, a large amount of textual information was retrieved from multiple web forums and social media platforms. The searches were conducted between February 7, 2022, and March 15, 2022. To avoid concerns about the accuracy and efficiency of the translation of a large number of posts, this study included only English-language texts.

For web forums, the top search engines globally, including Google, Microsoft Bing, Yahoo, and Yandex, were used to identify health forums using different combinations of search terms (“cancer,” “tumor/tumour,” “patient,” and “forum”) [23]. The search results were extracted and examined by 2 investigators. Web forums were included if (1) they appeared in more than one search result, (2) no membership or passwords were needed to access the messages, (3) the site had been active for >5 years, (4) at least 10 messages were posted to the group within the past 30 days from the date of the search, and (5) they enabled web scraping of posts or feeds using Python (Python Software Foundation) or R (R Foundation for Statistical Computing). Finally, 16 web-based health forums were included. The forums used in this study are listed in Table S2 in Multimedia Appendix 1.

For social media platforms, we extracted posts from Facebook, Twitter, and Reddit, which are among the most popular social networks and consist mainly of textual content [24]. As there were differences in the structure of the different social media platforms, the search strategies and selection criteria had to be adapted to each platform, as detailed in Table S3 in Multimedia Appendix 1.

### Data Collection and Extraction

The search keywords for TCIM were predefined with reference to multiple authoritative websites, such as the National Cancer Institute and the National Centre for Complementary and Integrative Health website (Table S4 in Multimedia Appendix 1). This study included a wide range of TCIM approaches to reflect real-life scenarios in which patients with cancer used diverse TCIM based on the literature [25,26]. All posts and comments were extracted from forums and social media platforms from their inception to February 7, 2022, using Python (except for Facebook Facepager) [27]. Only publicly available and anonymized data were collected, as real names were often unavailable and user IDs were not extracted. The posts and comments were then organized into databases for further processing.

### Data Preprocessing and Text Vectorization

Extracted textual data were preprocessed (removing punctuation, tokenization, lemmatization, stemming, and stop words) using NLP algorithms in the Python packages *Natural Language ToolKit* [28] and *SpaCy* [29]. NLP refers to automated machine-driven algorithms for semantic mapping, extracting information from, and understanding human language. It can be used to extract salient information from large amounts of unstructured text [22].

The processed text was organized into multiple databases for each TCIM modality and general category. We used the classification recommended by the National Centre for Complementary and Integrative Health, which grouped the modalities into 3 main categories: natural products; mind and body practices; and other complementary health approaches, including whole-system and alternative practices (Table S4 in Multimedia Appendix 1) [4].

### Descriptive and Sentiment Analysis

To explore the interest in TCIM shown by patients or the caregivers of patients who were diagnosed with different types of cancer or had various cancer-related symptoms, posts were further selected if they contained keywords related to the 10 most common cancer diagnoses globally and the most common cancer symptoms or treatment-related side effects (Table S5 in Multimedia Appendix 1).

Descriptive analyses were performed to explore the most commonly discussed TCIM modalities for each symptom and cancer type, according to the proportion of posts related to specific modalities. This study aimed to explore patients’ interest in and acceptance of various modalities in managing their symptoms. Sentiment analyses were performed to measure the polarity of each post or comment to determine how positive or negative the text was toward the use of TCIM or patient experience [21]. The Python library *TextBlob*, which actively uses the *Natural Language ToolKit* and lexicon-based approaches, was used in the analysis. It returned a polarity score for each post (lies between −1, which indicates a negative sentiment, and 1, which indicates a positive sentiment) that defines the sentiment [30]. Both positive and negative sentiment posts were summarized and classified into themes by 2 independent investigators. Positive themes included advantages and facilitators of using TCIM, whereas negative themes included disadvantages and barriers to using TCIM. Discrepancies were resolved by a third investigator.

### Exploratory Analysis (Topic Modeling)

Exploratory analyses were conducted using NLP methods to investigate the patients’ use and perceptions of TCIM for different symptoms. As there are diverse TCIM approaches, we were not able to deeply examine every approach. Instead, we adopted a more justifiable approach. Previously, we identified a priori some TCIM modalities that have been recommended in published integrative oncology guidelines or have emerging clinical evidence demonstrating its effectiveness in the past decade (cannabis, omega-3 fatty acids, acupuncture, acupressure, massage, hypnosis, yoga, and meditation) [31-33]. Unsupervised or semisupervised learning approaches have been used to perform topic modeling of posts containing keywords related to selected modalities or symptoms [34]. Topic modeling is a text-mining tool used to discover hidden semantic structures in a body of text by analyzing the occurrence of words in a collection of texts [22]. In this study, LDA, a probabilistic topic-modeling tool that groups or clusters discrete data such as a large set of texts based on the words that occur in them, was used [35]. We used Gensim for LDA topic modeling and displayed the top 50 keywords and their weights for each topic [36]. In unsupervised LDA models, the categorization of topics is based on NLP algorithms, including word frequency analysis, term frequency–inverse document frequency, or neural network-based analysis (eg, word2vec or Lda2vec) [22]. In semisupervised LDA models, a predefined set of keywords related to TCIM modalities that had previously been categorized was passed to the LDA model as seed information before performing topic modeling (Table S4 in Multimedia Appendix 1) [37]. Finally, the generated features were visualized in word clouds and reviewed by the investigators.

### Ethics Approval

This study was approved by the Survey and Behavioral Research Ethics Committee of the Chinese University of Hong Kong (reference SBRE-20-553).

## Results

### Overview

After extracting 6,120,063 posts from web forums and social media platforms, we removed duplicate (n=3,078,521) and non-English (n=1,420,787) posts or comments, leaving 1,620,755 posts available for analysis. Most of the forums originated in the United States or the United Kingdom (n=14) but were open to users in other countries.

### Descriptive Analyses of TCIM Use for Various Cancer Types and Symptoms

Table 1 lists the 5 most frequently mentioned symptoms for each cancer type in the extracted posts. Cancer-related symptoms, such as pain (10/10, 100% cancer types), anxiety and depression (9/10, 90%), poor sleep (9/10, 90%), and fatigue (8/10, 80%), are commonly discussed in most cancer types.

**Table 1:** Distribution of posts according to the types of cancer and common symptoms.

Symptom	Cancer type									
	Esophageal	Cervical	Liver	Stomach	Lung	Thyroid	Colorectal	Bladder	Prostate	Breast
Pain	Cannabis, fish oil, vitamin	Yoga^a^, chiropractor, cannabis	Cannabis, massage^a^, herb	Cannabis, supplement, massage^a^	Cannabis, acupuncture^a^, massage^a,b^	Massage^a^, acupuncture^a^, supplement	Cannabis, acupuncture^a^, supplement	Cannabis, supplement, massage^a^	Cannabis, supplements, vitamin	Acupuncture^a^, massage^a^, yoga^a^
Fatigue	N/A^c^	N/A	Cannabis, supplement, vitamin	Yoga, supplement, vitamin	Cannabis, acupuncture^a^, supplement	Calcium, vitamin, supplement	Acupuncture^a^, ginseng, vitamin	Cannabis, vitamin, supplement	Cannabis, qigong, yoga	Yoga^a^, acupuncture^a^, vitamin
Hot flash	N/A	Black cohosh, magnesium, acupuncture	N/A	N/A	N/A	N/A	N/A	N/A	N/A	N/A
Nausea or vomiting	Cannabis, ginger, fish oil	N/A	N/A	Cannabis, ginger, vitamin	N/A	N/A	Cannabis, ginger, acupuncture	N/A	N/A	N/A
Anxiety or depression	Cannabis, vitamin, meditation^a^	Meditation^a^, aromatherapy, cannabis	Cannabis, meditation^a^, herb	N/A	Cannabis, magnesium, yoga^a^	Yoga^a^, meditation^a^, vitamin	Fish oil, cannabis, vitamin	Cannabis, meditation^a^, Mediterranean (diet)	Vitamin, meditation^a^, cannabis	Yoga^a^, meditation^a^, massage
Diarrhea or constipation	N/A	N/A	N/A	N/A	N/A	N/A	Probiotics, supplement, ginger	N/A	N/A	N/A
Cough	N/A	N/A	N/A	N/A	Cannabis, herb, breathing (exercises)	N/A	N/A	N/A	N/A	N/A
Loss of appetite	Cannabis, fish oil, supplement	N/A	Cannabis, supplement, herb	Cannabis, supplement, fish oil	N/A	N/A	N/A	N/A	N/A	N/A
Edema	N/A	N/A	N/A	N/A	N/A	Massage, acupuncture, yoga	N/A	N/A	N/A	Yoga, massage, acupuncture
Infection or neutropenia	N/A	Vitamin, active hexose correlated compound, green tea	N/A	N/A	N/A	N/A	N/A	Cranberry, Newcastle (virus), vitamin	N/A	N/A
Memory loss	N/A	N/A	N/A	N/A	N/A	N/A	N/A	N/A	Supplement, pomegranate, vitamin	N/A
Insomnia or poor sleep	Melatonin, cannabis, supplement	Melatonin, meditation^a^, supplement	Cannabis, supplement, melatonin	Cannabis, massage, yoga	Cannabis, melatonin, massage	Acupuncture, melatonin, supplement	N/A	Cannabis, melatonin, supplement	Cannabis, melatonin, meditation^a^	Melatonin, yoga^a^, massage

^a^Modalities with suggested indications that are included in the integrative medicine guidelines.

^b^The top 3 traditional, complementary, and integrative medicine modalities for the top 5 symptoms most frequently discussed in each cancer type are listed.

^c^N/A: not applicable.

There were some similarities in the most frequently discussed TCIM modalities used to treat symptoms across various cancer types. For instance, cannabis was among the most frequently discussed TCIM modalities for pain in 7 (70%) of the 10 cancer types. Cannabis was also among the most frequently mentioned TCIM modalities for several other symptoms including nausea and vomiting (3/3, 100%), loss of appetite (3/3, 100%), anxiety and depression (7/9, 78%), and poor sleep (6/9, 67%). Meditation was the most frequently discussed TCIM modality for anxiety and depression (7/9, 78%), whereas melatonin (8/9, 89%) was commonly mentioned for poor sleep. However, there are no dominant TCIM modalities for fatigue in different types of cancer. The 5 most frequent TCIM terms in the posts for each cancer-related symptom are listed in Table S6 in Multimedia Appendix 1.

### Thematic Analyses of TCIM Use and Perceptions

A total of 7 positive and 7 negative themes were identified; examples of quotes for each theme are listed in Tables 2 and 3.

**Table 2:** Positive themes and notable quotes from the posts.

Positive themes	Quotes
Make symptoms manageable	“(Cannabidiol/medical marijuana) do not kill the pain like opioids but they make it (the pain) manageable.” “Does it (cannabidiol) cure anything? No. But for pain, anxiety, or sleep issues? I am sure the relief some get from it is a miracle to them.” “Acupuncture helped to resolve my back pain, and allowed me to become more mobile. which meant I could walk and exercise more...”
Benefit mind and body	“(Yoga or stretching) it is giving me a real love for my body and mind and spirit I never experienced before.” “Meditation also gets my mind focused when I need to have a clear head for work.”
Reduce the need for prescription medications	“...have had some relief (of insomnia) with time released melatonin, magnesium supplements and valerian-trying to avoid prescription sleep meds." "I already turned down her offer of Effexor to combat hot flashes. This drug will put me into permanent menopause, increases my risks of ovarian and uterine cancers and bone loss. I have been detoxing my environment...and taking supplements supported by the Gerson therapy.”
Provide options when intolerant of conventional treatment	“I was seriously considering going off Arimidex because I increasingly becoming so achy...I read that acupuncture helps with the joint pains. I thought I should give it try.” “I am allergic to most all pain drugs, I finally resorted to acupuncture.” “I really do not want to go on an antidepressant since it is contraindicated with tamoxifen...I read a study on magnesium and depression so I have upped my supplement.”
Reduce the need and side effects from conventional cancer therapies	“Surgery to remove the stomach is imminent though. but hopefully, the acupuncture and the sjw tea will keep chemo and radiation at bay...” “The cannabidiol is the only thing that lessens the painful side effects of his drugs.” “I really believe that all this has helped me manage side effects, reduce tiredness, rebound to feeling “normal” again.”
Nonexclusive nature of traditional, complementary, and integrative medicine and conventional therapies	“I believe in natural treatments, too. but, not to the exclusion of a doctor’s...there’s nothing wrong with using both treatments and not excluding one.” “Good for you both for looking into both allopathic and alternative (I call them competitive) medical approaches. They are not mutually exclusive.”
Self-perceived effectiveness	“I'm of the view if you think something is doing you good and working in your situation, then it probably is. So if you think acupuncture will help then it probably does.”

**Table 3:** Negative themes and notable quotes from the posts.

Negative themes	Quotes
Side effects and undesirable symptoms from TCIM^a^	“Cannabidiol gave him awful pain and an acrid feeling in his mouth and throat.” “(Honey) may contain bacteria that could increase the risk of infection to someone with a lowered immune system.” “When platelets were falling, that fish oil and (vit) e tend to thin the blood a bit...” “She tried acupuncture for pain management and that made her so much worse to the point where she cannot do basic household chores.”
Risk of interactions between TCIM and existing treatment	“There is a theoretical risk that manuka honey can lessen the effect of some chemotherapy drugs.” “The radioactive iodine treatment requires you to be pretty solitary for a week...and do not take a multivitamin with iodine in it! that was my big mistake.”
Refusal of conventional treatments because of TCIM	“She refused all conventional treatment, including pain relief, and blamed herself for not being able to effect a cure with “natural medicine.” “A friend was dying of breast cancer last year and walked away from all professional treatments for a diet therapy from the internet against medical advice...it claimed radical cure rates and much better responses than chemo.”
View TCIM as nonscientific or the lack of evidence	“I truly loathe pseudo science! all types of people coming out of the woodwork with “advice” everything from consuming raw ginger to cannabidiol to bleeding!” “Websites tout (cannabis) as not just soothing, but curative; an alternative for chemo even. There is no evidence for this.”
Delayed diagnosis and treatment because of TCIM	“My wife was diagnosed for 7 mo with a pulled chest muscle that turned out to be lung cancer, she had no x-rays or scans or other treatment and was advised to see chiropractor as you can imagine caused more pain than necessary.”
Doctors’ ignorance of patients’ interest in TCIM and their use	“The doctors will not believe me so I am having acupuncture which is really good.” “They are not mutually exclusive, although many allopathic doctors hate the competition...” “My oncologist did not want me to take anything, but I decided to take the cannabidiol oil and it worked.”
Lack of information from doctors	“Many docs do not really provide much information on the importance of diet, nutrition, and spiritual/psychological wellness.”

^a^TCIM: traditional, complementary, and integrative medicine.

Positive themes about TCIM included making symptoms, such as pain and anxiety, more manageable for patients; providing benefits to the mind and body; and reducing the need for prescription drugs, such as sleep and pain medications. TCIM have also been reported to reduce the side effects of medications. Concerns with the side effects, allergies, or drug interactions associated with conventional treatments are one of the facilitators of TCIM use, as it offers an alternative option for these patients. Another facilitator was the patient’s view that TCIM and conventional treatments were not considered mutually exclusive and could be used together.

Conversely, some posts expressed concerns about TCIM use, including undesirable effects (such as pain and bleeding from taking certain supplements) and potential risks of interaction with conventional medications. Moreover, some viewed TCIM as nonscientific or not evidence-based, while stating that it caused the refusal of conventional treatments or delays in the diagnosis and treatment of some patients, leading to unnecessary distress and reduced survival. These perceptions may prevent them from considering TCIM. Other barriers to TCIM use were centered on doctors’ ignorance and a lack of information about TCIM.

### Exploratory Analyses

In the exploratory analysis, we identified agreements between the themes from topic modeling and the above descriptive analyses regarding TCIM modalities for cancer-related symptoms (Figure S1 in Multimedia Appendix 1). For example, vitamins and supplements were frequently mentioned in posts about anemia, yoga and meditation were mentioned in posts about anxiety and depression, and massage was mentioned in posts about lymphedema.

For TCIM modalities that are emerging or have been recommended in recent guidelines, topic modeling revealed some meaningful topics in the discussions (Table S7 in Multimedia Appendix 1 and Multimedia Appendix 2). The most common theme was TCIM modalities that may be used to manage symptoms, for example, cannabis for pain, anxiety, nausea, appetite, and poor sleep; acupressure for nausea, fatigue, and pain; acupuncture for nausea and vomiting, lymphedema, pain, hot flashes, and neuropathy; meditation for poor sleep, pain, anxiety, and depression; yoga for pain, weight change, poor sleep, anxiety, fatigue, and lymphedema; hypnosis for pain, anxiety, and poor sleep; omega-3 fatty acids for pain and weight change; and massage for poor sleep, pain, neuropathy, lymphedema, and fatigue.

Moreover, the visualization of topic modeling revealed other notable themes in the discussion (Table S7 in Multimedia Appendix 1 and Multimedia Appendix 2). For cannabis, concerns about its legalization and prohibition of use were raised (“legalize,” “legal,” “prohibition,” “strain,” “access,” and “state”). Techniques for self-administration were also frequently mentioned in acupressure posts (“technique,” finger, “point,” “wrist band,” and “pressure”). TCIM may also provide extra benefits, such as positive experiences (meditation—“cope,” “healing,” “relax,” “positive,” and “hope”) and rehabilitation (yoga—“recovery,” “improve,” “strength,” “heal,” and “restorative”). Notably, “cure” appeared in some visualizations for certain modalities, including cannabis and acupressure.

## Discussion

### Principal Findings

Several evidence-based guidelines on integrative oncology have been established, including integrative pain management and the use of complementary therapies for breast and lung cancers [31-33]. By exploring web-based discussions on TCIM use from multiple health forums and social media platforms, we found that many of these recommended therapies and their respective indications, such as acupuncture for pain and yoga for anxiety, were commonly discussed by patients and their caregivers. In addition, we were able to identify other TCIM modalities that are popular among the web-based cancer community for managing symptoms. Several positive and negative themes related to TCIM were identified, showing the facilitators of and barriers to TCIM use and the importance of patient-physician communication regarding TCIM. For example, the lack of information from doctors may prevent patients from considering using TCIM. Emerging evidence supports the use of certain TCIM modalities, such as acupuncture for cancer pain and meditation for anxiety or stress reduction, which, coupled with the growing awareness and acceptance of TCIM, reflect the increasing importance of integrating TCIM therapies into routine cancer care [31,33]. Our study also showed the great potential of leveraging patient-generated data from the internet and providing directions for future research on TCIM and its integration into cancer care. For example, we found great interest from patients using cannabis in relieving cancer-related symptoms, which warrants further clinical research. As there are many TCIM practices, research in integrative oncology requires an understanding of patient preferences, values, and receptivity from real-world data sources, one of which is the internet, to inform research priorities [38]. The facilitators and barriers identified in this study can also facilitate the integration of evidence-based TCIM practices into routine practices, as well as incorporating patient preferences into clinical practice guidelines [39,40].

One important theme identified from the study was patients’ views of the role of TCIM and conventional therapies as complementary to each other, instead of being mutually exclusive, as previously thought [41,42]. During active cancer treatment and the survivorship phase, patients may have a high symptom burden and often require multiple medications [43]. Polypharmacy is associated with a lower quality of life and increased risk of adverse effects [44,45]. In this study, patients pointed out that TCIM may help reduce the side effects of medications and the need for supportive medications. This finding is consistent with previous surveys showing that managing the complications of cancer or its therapies is one of the major reasons for TCIM use [2]. Another notable role of TCIM, which has been less commonly mentioned in previous studies, was to provide an alternative when conventional supportive treatment was not tolerated. This is important because the symptom burden can significantly decrease the quality of life of patients and survivors of cancer [46]. Overall, our results suggest that TCIM may fill gaps in cancer symptom management and that the integration of TCIM into routine cancer care is justified. TCIM modalities should be used in conjunction with conventional therapies (such as medications and surgery) as integrative medicine to maximize the benefits of both approaches in patients with cancer.

Our study identified several barriers to effective communication regarding the TCIM. For instance, ignorance by doctors regarding TCIM use or symptoms experienced by patients has been reported. Effective incorporation of integrative therapies into oncology care requires communication between patients, caregivers, and doctors. However, previous studies have found that some patients do not disclose their use of TCIM because they think that their doctors consider TCIM to compete with conventional treatment, they are not aware of the potential interaction between TCIM and conventional medicines, or because clinicians did not initiate discussions on TCIM during the consultations [47,48]. This suggests the need for open-mindedness among doctors about integrative therapies and effective communication about TCIM between doctors and patients. For instance, doctors can recognize potential TCIM options by initiating conversations regarding TCIM during consultations when discussing treatment-related side effects or other symptoms experienced by patients. Studies have also found that discussing TCIM can enhance patient-physician relationships [49]. From the doctors’ perspective, one of the major reasons for not initiating TCIM-related discussions is the inadequacy of medical training in this area [50]. Therefore, oncology-specific TCIM training should be provided to physicians to fill this knowledge gap. Currently, there has been an increasing effort to develop integrative oncology education programs and competencies, such as knowledge of the safety and effectiveness of TCIM [51,52]. In the future, enhancing the training of doctors in integrative oncology could increase their confidence in communicating with patients regarding the use of TCIM. Moreover, tailored communication training can be provided to all oncology practitioners, conventional or TCIM, to promote patient-centered communication on integrative medicines. Referral pathways should also be established for patients who opt for specialized TCIM advice.

Notably, concerns were raised on social media platforms and forums that some patients with cancer who used TCIM chose to forgo conventional treatment, causing delays in the diagnosis and treatment of cancer. A previous study showed that TCIM use reduced survival in patients with cancer and found that the major reason for this result was their refusal of conventional cancer treatment and low treatment adherence [53]. To avoid delays in appropriate treatments, doctors and TCIM professionals should be more proactive in examining the needs and concerns of patients and, if possible, provide evidence-based recommendations on integrative medicines to patients for symptom management. This can be achieved by establishing multidisciplinary teams comprising doctors, TCIM professionals, and other health care professionals. Although primary oncologists play a major role in communicating with patients regarding the treatment progress and assessing their symptom burden, they can refer patients who are interested in using other approaches to TCIM professionals based on their preferences. This allows interdisciplinary communication between different health care professionals to provide tailored regimens, including conventional therapies and TCIM. To enhance the effectiveness of communication, doctors and TCIM professionals can consider web-based joint consultations with patients to discuss their holistic care plans throughout their cancer care continuum, especially for patients and health care professionals residing or working in distant locations [54,55].

Although there is growing clinical evidence for the efficacy of some TCIM modalities, patients also use a wide range of other TCIM modalities that have been less frequently studied. In this study, NLP methods were used to reveal patients’ use of different TCIM modalities for managing a variety of cancer-related symptoms. In particular, our results suggest a strong interest from patients regarding the use of cannabis, although concerns about its legality of use were common in the discussions. As of 2022, approximately 70 countries have permitted some form of medical cannabis use [56]. Indeed, there has been a heated debate on the legalization of cannabis, especially in response to opioid crises [57]. Patients and their caregivers mostly discussed the use of cannabis for the management of various physical symptoms, such as pain and gastrointestinal symptoms, which was consistent with findings from previous surveys in the United States and Canada [58,59]. Patients also discussed the use of cannabis for neuropsychiatric symptoms, such as anxiety and poor sleep. Despite the popularity of cannabis, evidence for its therapeutic effect remains scant [60,61], and more research is necessary to support its use in supportive care, especially for psychological symptoms. Meanwhile, proactive initiation of communication with patients on the use of cannabis by doctors may be appropriate, considering the immense interest from patients and potential concerns, including its adverse effects and interactions.

The strength of our study is that a large number of posts from multiple health forums and social media platforms were extracted for analysis using machine learning techniques. We also generated greater insights into TCIM use based on different cancer types, common symptoms, and specifically recommended modalities by guidelines and emerging modalities that have not been well studied previously using web-based patient-reported data [31-33]. However, this study mostly identified the patients’ interests and concerns among the web-based cancer community and may be less representative of patients with cancer who were not forums or social media users, especially the older population. Moreover, only English-language posts were extracted for analysis, and the selected forums and social media platforms did not include those using non-English languages as the medium of communication, as we only used search terms in English to extract the websites. The patterns of TCIM use and views on TCIM may vary across different cultures. For instance, traditional Chinese medicine and Ayurveda are common in Chinese and Indian communities, respectively, but less common in Western countries [62]. Cannabis is more commonly used legally in the United States and Europe, yet it is largely restricted in many Asian countries [56,63]. Therefore, future studies should consider including posts in other languages and from other forums and social media platforms to explore the differences in interests and concerns about TCIM across countries and cultures. The differences in TCIM-related content among web-based platforms can also be compared. Furthermore, the limitations of sentiment analyses using machine learning approaches may result in the misclassification of posts into positive and negative sentiments; for example, failure to identify sarcasm and polarity classification can vary across different topics [64]. Nevertheless, we supplemented the analyses with manual verification before summarizing the themes from the posts to minimize errors. Although we acknowledge that popularity does not equate to effectiveness, findings from this study can inform future research in integrative oncology based on patients’ interests and the popularity of TCIM approaches.

### Conclusions

This study showed that patients with cancer and their caregivers used social media and health forums to express their opinions and share their experiences regarding TCIM use. Patients commonly discuss the use of TCIM, especially cannabis and certain mind-body therapies, for cancer-related symptoms. Facilitators and barriers to TCIM use were identified from discussions. The results suggest that effective communication about TCIM should be achieved to help understand patients’ concerns and that doctors should be more open-minded to actively discuss TCIM use with their patients. Machine learning approaches, such as NLP, are promising tools for generating useful information regarding the pattern of use and patients’ perceptions of TCIM from patient-generated data on health forums and social media platforms. These approaches may also provide directions for future research into TCIM modalities and their uses, which are most popular among patients. Future studies should also use these methods to explore the use of and interest in using TCIM for other chronic diseases.

## Appendix

Multimedia Appendix 1 List of web-based health forums, social media platforms; keywords related to traditional, complementary, and integrative medicine; symptoms; cancer diagnoses; and selected words with high weights related to topics categorized by topic modeling.

Multimedia Appendix 2 Visualizations of exploratory analysis of themes on traditional, complementary, and integrative medicine modalities (selected).

## Abbreviations

LDA: latent Dirichlet allocation
NLP: natural language processing
TCIM: traditional, complementary, and integrative medicine

## Data Availability

All data generated or analyzed during this study are included in this published article and its supplementary information files.
