# Supplementary material for: The Use of Traditional, Complementary, and Integrative Medicine in Cancer: Data-Mining Study of 1 Million Web-Based Posts From Health Forums and Social Media Platforms
Source: J Med Internet Res. 2023 Apr 21;25:e45408. doi: 10.2196/45408 (PMC10163397; doi:10.2196/45408)
Supplement: Multimedia Appendix 1 [file jmir_v25i1e45408_app1.docx]

**Table S1. Definitions and Examples of Traditional, Complementary and Integrative medicine (TCIM)**

| **Terms** | **Definitions** | **Examples** |
| --- | --- | --- |
| **Traditional medicine** | The sum total of the knowledge, skill and practices based on the theories, beliefs and experiences indigenous to different cultures, whether explicable or not, used in the maintenance of health as well as in the prevention, diagnosis, improvement or treatment of physical and mental illness [1] | - Traditional Chinese medicine  - Ayurvedic medicine  - Unani medicine  - Tibetan medicine |
| **Complementary medicine** | A broad set of health care practices that are not part of that country’s own traditional or conventional medicine and are not fully integrated into the dominant health care system. They are used interchangeably with traditional medicine in some countries [1] | - Dietary supplements  - Homeopathy  - Chiropractic  - Acupuncture |
| **Integrative medicine** | Integrative medicine brings conventional and complementary approaches together in a coordinated way. It also emphasizes multimodal interventions, which are two or more interventions such as conventional health care approaches and complementary health approaches in various combinations [2,3] | - Conventional healthcare approaches (e.g., medication, physical rehabilitation) + Complementary health approaches (e.g., acupuncture, yoga, probiotics) in various combinations |

**References:**

[1] World Health Organization. Traditional, complementary and integrative medicine. Available at: http://www. who. int/traditional-complementary-integrative-medicine/en. (Accessed Dec 14, 2022)

[2] National Center for Complementary and Integrative Health. Complementary, Alternative, or Integrative Health: What’s In a Name? Available at: https://www.nccih.nih.gov/health/complementary-alternative-or-integrative-health-whats-in-a-name. (Accessed on Dec 14, 2022)

[3] Adams, J. (2019). Public Health and Health Services Research in Traditional, Complementary and Integrative Health Care: International Perspectives. World Scientific.

**Table S2. List of online health forums selected in the data extraction**

| Name of web forums | URL | Originating countries |
| --- | --- | --- |
| Cancer Chat | https://www.cancerresearchuk.org/about-cancer/cancer-chat | UK |
| Jo's Cervical Cancer Trust Forum | https://www.jostrust.org.uk/forum | UK |
| Prostate Cancer UK | https://community.prostatecanceruk.org/alltopics | UK |
| Bowel Cancer UK Community | https://community.bowelcanceruk.org.uk/forum/ | UK |
| Cancer Survivors Network | https://csn.cancer.org/forum | US |
| Breastcancer.org community | https://community.breastcancer.org/ | US |
| Pancreatic Cancer UK Forum | https://forum.pancreaticcancer.org.uk/ | UK |
| Breast Cancer Now Forum | https://forum.breastcancernow.org/ | UK |
| Cancer Council Online Community | https://onlinecommunity.cancercouncil.com.au/ | Australia |
| Irish cancer society community | https://www.cancer.ie/community | Ireland |
| Navigating Care | https://www.navigatingcare.com/explore/all/discussions | US |
| TC Cancer.com | http://www.tc-cancer.com/forum/# | US |
| HealthBoards (cancer) | https://www.healthboards.com/boards/cancers/ | US |
| Melanoma Patient Forum | https://forum.melanoma.org/ | US |
| Macmillan Cancer Support | https://community.macmillan.org.uk/g | UK |
| Healthunlocked | https://healthunlocked.com/search/communities?query=cancer | UK |

**Table S3. List of social media platforms selected in the data extraction and the search process**

| 1. Facebook |  |
| --- | --- |
| Types and characteristics as social media | Social networking sites: Web-based services that allow individuals to construct a public or semi-public profile within a bounded system, (2) articulate a list of other users with whom they share a connection, and (3) view and traverse their list of connections and those made by others within the system |
| Search process and criteria | The Facebook search engine function was used to identify Facebook pages for analysis using the search terms (“cancer”, “tumor/tumour”, “patient”, “forum” or other related terms in English). The search results were restricted to pages and groups that are publicly available. The first 25 results were selected and screened, and relevant pages/groups that contain >1,000 members were included. |
| Examples | Cancer Sucks (US), Indian Cancer Society (India), Health Forum with Doc Atoie (Philippines), Campaigning for Cancer (South Africa), Cancer Support Community (Global) |
| 2. Reddit |  |
| Types and characteristics as social media | Social news: Services that allow people to post various news items or links to outside articles and then allows its users to “vote” on the items. The voting is the core social aspect as the items that get the most votes are displayed the most prominently. The community decides which news items get seen by more people. |
| Search process and criteria | Threads were searched using a systematic approach. First, subreddits that were related to cancer and had >1000 members were selected, and threads were then extracted from each subreddit by searching relevant terms or words. Keywords related to the TCIM that were used in the thread search are shown in Supplementary Table 3. Selected threads included the original post and comments/responses. |
| Examples | r/cancer, r/breastcancer, r/lymphoma, r/CancerFamilySupport, r/Prostatecancer, r/Cancersurvivors |
| 3. Twitter |  |
| Types and characteristics as social media | Microblogging: Services that focus on short updates that are pushed out to anyone subscribed to receive the updates |
| Search process and criteria | Search was conducted on publicly available user profiles on Twitter. We examined the number of user accounts in which the names of cancers are described in the profile. The search terms included those relate to cancer (cancer, tumor/tumour, lymphoma, leukemia) and TCIM (Supplementary Table 3). |

References:

McCay-Peet, L., & Quan-Haase, A. (2017). What is social media and what questions can social media research help us answer. *The SAGE handbook of social media research methods*, 13-26.

Eghtesadi, M., & Florea, A. (2020). Facebook, Instagram, Reddit and TikTok: a proposal for health authorities to integrate popular social media platforms in contingency planning amid a global pandemic outbreak. *Canadian Journal of Public Health*, 111, 389-391.

**Table S4. Example of keywords related to TCIM for post/thread search and the data sources**

| **Keywords** |
| --- |
| **Natural Products**  beta-carotene, biotin, folic, vitamin, calcium, chromium, magnesium, tocopherol, ascorbic, selenium, zinc, boneset, acai, agaricus, AHCC, alfafa, aloe, andrographis, angelica, apricot, arnica, artemisia, artemisinin, ashwagandha, astragalus, bilberry, bitter-melon, bitter-orange, black-cohosh, botanic, burdock, butterbur, calendula, cannabis, capsaicin, carotene, cascara, cat’s-claw, chaga, chamomile, chaste, chia, chrysanthemum, cinnamon, cohosh, cordyceps, cranberry, dandelion, devil’s-claw, echinacea, elderberry, ellagic, emblica, fenugreek, feverfew, flax, forskolin, frankincense, fucoidan, garcinia, garlic, ginger, ginkgo, ginseng, graviola, green-tea, guarana, herb, kelp, linseed, oleander, primrose, remifemin, spirulina, squawroot, wormwood, yunzhi, clover, ganoderma, glycyrrhiza, goji, goldenseal, goldthread, gotu-kola, grapeseed, hawthorn, hervea, hoodia, hops, horse-chestnut, kava, kratom, kudzu, lavender, lemongrass, lentinan, licorice, maca, milk-thistle, mistletoe, mulberry, myrrh, nigella, noni, oleandrin, passionflower, pectin, pelargonium, peppermint, propolis, pygeum, quercetin, raspberry, redbush, resveratrol, rhodiola, rhubarb, sativa, shitake, slippery-elm, viscum, wolfberry, yerba, eleuthero, saw-palmetto, st-john’s-wort, salvia, tea-tree, turmeric, curcumin, valerian, wild-yam, yohimbe,alpha-lipoic, amygdalin, arginine, biobran, bromelain, carnitine, cesium, chitosan, fish-oil, germanium, glucosamine, glutamine, linoleic, limonene, lipoic, prebiotic, probiotic, q10, supplement, dha, inositol, leucine, lutein, lycopene, manuka, melatonin, natto, omega, soy |
| **Mind and body practices**  acupressure, acupuncture, electroacupuncture, acupoint, alexander, biofeedback, chiropractic, chiropractor, Feldenkrais, hydrotherapy, hypnotherapy, hypnosis, imagery, manipulation, massage, meditation, transcendental, mind-body, osteopath, pilate, prayer, qigong, reflexology, reiki, relaxation, rolfing, shiatsu, spirituality, taichi, trager, visualization, yoga, exercise, cognitive-behavioral, meridian, moxibustion, breathwork, craniosacral, myotherapy, myofascial, tuina, bodywork, eurythmy, energy-healing, bioenergetic, therapeutic-touch, mindfulness, music-therapy, healing-touch, art-therapy, cupping |
| **Other complementary health approaches (whole-system approaches/alternative practices)**  chinese-medicine, TCM, oriental, anthroposophic, ayurveda, homeopath, naturopath, unani, arabic-medicine, kampo, Tibetan,  budwig, gerson, Kelley, Gonzalez, macrobiotic, immuno-augmentation, Newcastle, antineoplaston, di-bella, hoxsey, cancel, protocell |
| **Data sources** |
| Cancer Research UK  <https://www.cancerresearchuk.org/about-cancer/cancer-in-general/treatment/complementary-alternative-therapies/individual-therapies>  National Centre for Complementary and Integrative Health  <https://www.nccih.nih.gov/health/complementary-alternative-or-integrative-health-whats-in-a-name>  World Health Organization  <https://www.who.int/traditional-complementary-integrative-medicine/WhoGlobalReportOnTraditionalAndComplementaryMedicine2019.pdf>  Memorial Sloan Kettering Cancer Centre  <https://www.mskcc.org/cancer-care/diagnosis-treatment/symptom-management/integrative-medicine/herbs> Drexel University Library  <https://libguides.library.drexel.edu/c.php?g=176936&p=1389903>  National Cancer Institute  <https://cam.cancer.gov/health_information/cam_therapies_a-z.htm> |

**Table S5. 10 most common cancer diagnoses globally and the most common cancer symptoms or treatment-related side effects**

| Top 10 cancer diagnoses |
| --- |
| Breast  Lung  Colorectal  Prostate  Stomach  Liver  Cervix uteri  Oesophagus  Thyroid  Bladder  Data sources:  World cancer research fund (<https://www.wcrf.org/cancer-trends/worldwide-cancer-data/>)  Global cancer observatory (https://gco.iarc.fr/) |
| Common cancer-related symptoms |
| Pain, Fatigue, Hot flash, Nausea/ Vomiting, Hiccup, Xerostomia, Neuropathy, Anxiety/ Depression, Urination difficulties, Bleeding, Diarrhea/ Constipation, Cough, Loss of appetite, Night sweat/Fever, Headache, Seizure, Weight gain/ loss, Anemia, Edema, Infertility, Alopecia, Infection/ Neutropenia, Memory loss, Insomnia/ Poor sleep  Data sources:  NIH (<https://www.cancer.gov/about-cancer/diagnosis-staging/symptoms>)  Cancer Research UK (<https://www.cancerresearchuk.org/about-cancer/cancer-symptoms>)  CDC (<https://www.cdc.gov/cancer/survivors/patients/side-effects-of-treatment.htm>) |

**Table S6. Top 3 TCIM modalities among the posts for each common cancer-related symptom**

| Cancer-related symptoms | Top 3 TCIM modalities among the posts |
| --- | --- |
| Pain | massage, cannabis, supplement |
| Fatigue | supplement, yoga, acupuncture |
| Hot flash | black cohosh (herb), magnesium (supplement), acupuncture |
| Nausea/vomiting | vitamin, ginger (herb), cannabis |
| Hiccup | vitamin, ginger (herb), acupuncture |
| Xerostomia | acupuncture, vitamin, ginger (herb) |
| Neuropathy | B6 (vitamin), glutamine (supplement), acupuncture |
| Anxiety/Depression | cannabis, meditation, vitamin |
| Urination difficulties | vitamin, supplement, cranberry (herb) |
| Bleeding | vitamin, massage, arnica (herb) |
| Diarrhea/ Constipation | ginger (herb), magnesium (supplement), probiotic (supplement) |
| Cough | supplement, vitamin, ginger (herb) |
| loss of Appetite | cannabis, supplement, vitamin |
| Night sweat/Fever | vitamin, supplement, massage |
| Headache | vitamin, supplement, massage |
| Seizure | cannabis, vitamin, supplement |
| Weight gain/ loss | vitamin, supplement, yoga |
| Anemia | B12 (vitamin), vitamin, supplement |
| Edema | massage, yoga, vitamin |
| Infertility | vitamin, acupuncture, herb |
| Alopecia | biotin (vitamin), calcium (supplement), massage |
| Infection/ Neutropenia | vitamin, supplement, cranberry (herb) |
| Memory loss | vitamin, supplement, turmeric (herb) |
| Insomnia/ Poor sleep | melatonin (supplement), supplement, cannabis |

**Table S7. Selected words with high weights related to topics categorized by Latent Dirichlet allocation topic modeling for each traditional, complementary, and integrative medicine modality**

| **Acupressure** | **Proportion (%)^a^** | **Omega** | **Proportion (%)^a^** | **Acupuncture** | **Proportion (%)^a^** | **Cannabis** | **Proportion (%)^a^** |
| --- | --- | --- | --- | --- | --- | --- | --- |
| Acupressure | 100 | Fish | 98.8 | Acupuncture | 100 | Cannabis | 86.9 |
| Treatment | 84.9 | Help | 81.4 | Treatment | 100 | Hemp | 50.2 |
| Nausea | 76.4 | Omega | 79.0 | Nausea | 38.6 | Cure | 43.7 |
| Breast | 61.8 | Pain | 70.8 | Point | 37.5 | Marijuana | 41.0 |
| Fatigue | 59.3 | Food | 64.2 | Needle | 31.4 | Pain | 18.0 |
| Pain | 50.6 | Chemo | 57.4 | Lymphedema | 31.4 | State | 7.9 |
| Band | 40.7 | Diet | 54.7 | Pain | 29.8 | Legal | 6.4 |
| Chemo | 40.0 | Supplement | 48.1 | Healing | 27.3 | Prohibition | 5.6 |
| Wrist | 27.3 | Joint | 39.6 | Vomiting | 23.9 | Anxiety | 5.6 |
| Point | 26.4 | Weight | 21.5 | Flash | 17.6 | Nausea | 4.5 |
| **Meditation** | **Proportion (%)^a^** | **Hypnosis** | **Proportion (%)^a^** | **Yoga** | **Proportion (%)^a^** | **Massage** | **Proportion (%)^a^** |
| Meditation | 100 | Hypnosis | 99.2 | Yoga | 100 | Massage | 100 |
| Help | 100 | Pain | 81.9 | Pain | 86.2 | Chemo | 55.6 |
| Sleep | 46.2 | Smoke | 78.8 | Chemo | 67.9 | Breast | 50.4 |
| Mindfulness | 42.2 | Hypnotherapy | 66.4 | Exercise | 59.9 | Foot | 47.8 |
| Chemo | 39.1 | Lung | 23.0 | Weight | 55.2 | Therapy | 45.0 |
| Pain | 38.9 | Breast | 21.0 | Surgery | 53.9 | Sleep | 36.1 |
| Anxiety | 35.9 | Relief | 19.8 | Support | 42.4 | Support | 27.0 |
| Mind | 35.4 | Chemo | 19.2 | Sleep | 27.7 | Pain | 21.2 |
| Hope | 31.3 | Hope | 12.8 | Muscle | 27.0 | Neuropathy | 15.5 |
| Positive | 21.0 | Anxiety | 10.4 | Anxiety | 15.1 | Lymphedema | 14.2 |

^a^The proportion (%) refers to the percentage of posts containing the word among all posts related to a particular topic categorized by Latent Dirichlet allocation topic modeling. The proportion has been adjusted by weighting as the weights are according to different topics categorized by LDA topic modeling.


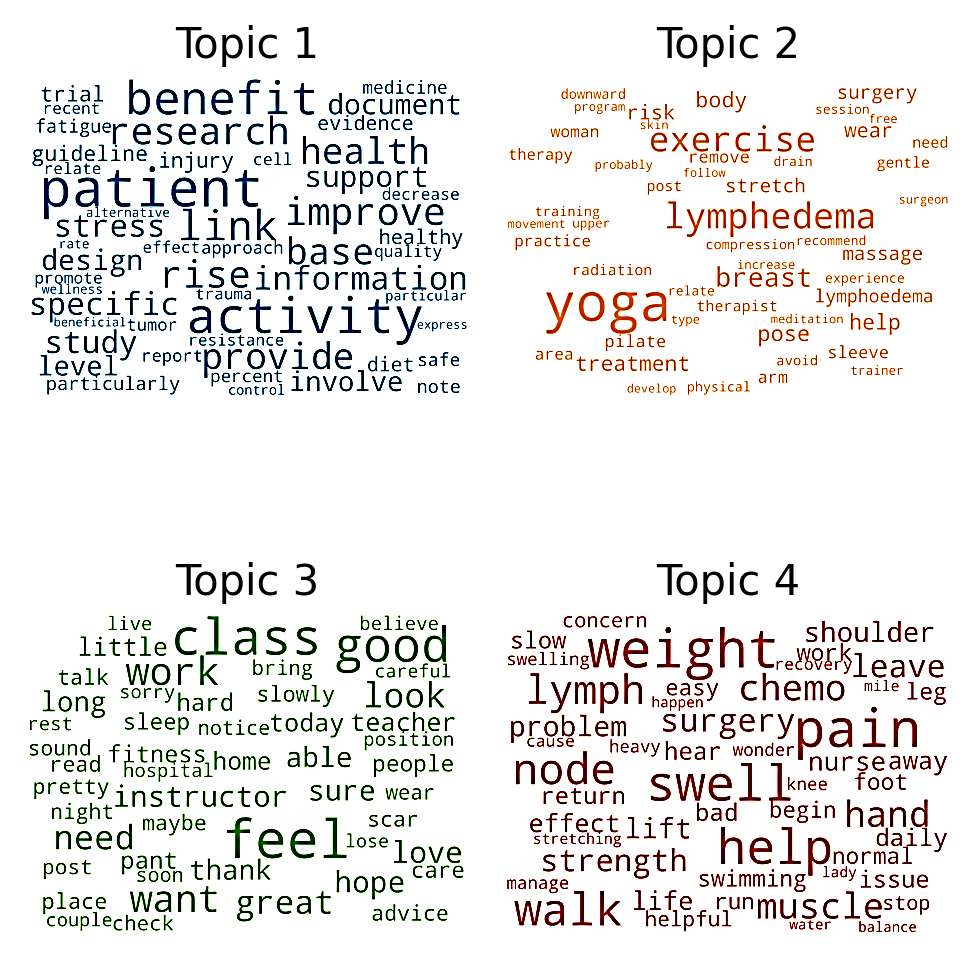
**
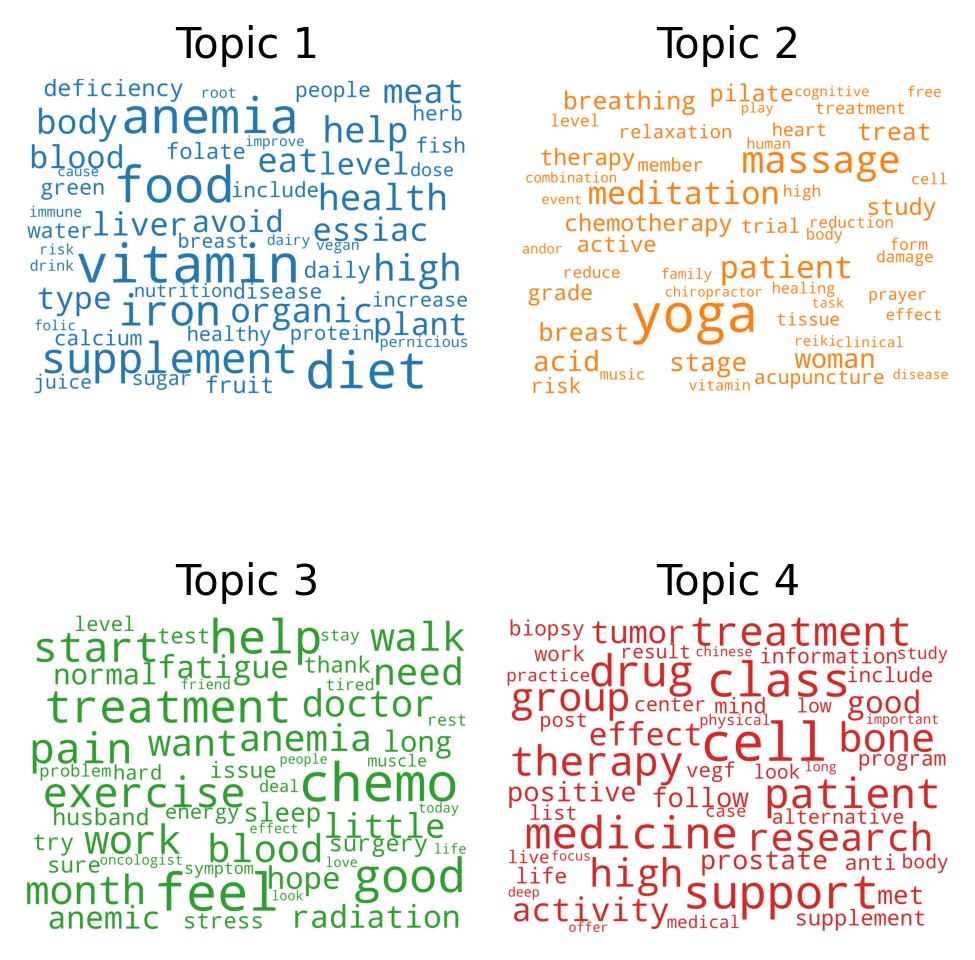
Figure S1. Selected wordclouds by topic modelling (symptom-based)**


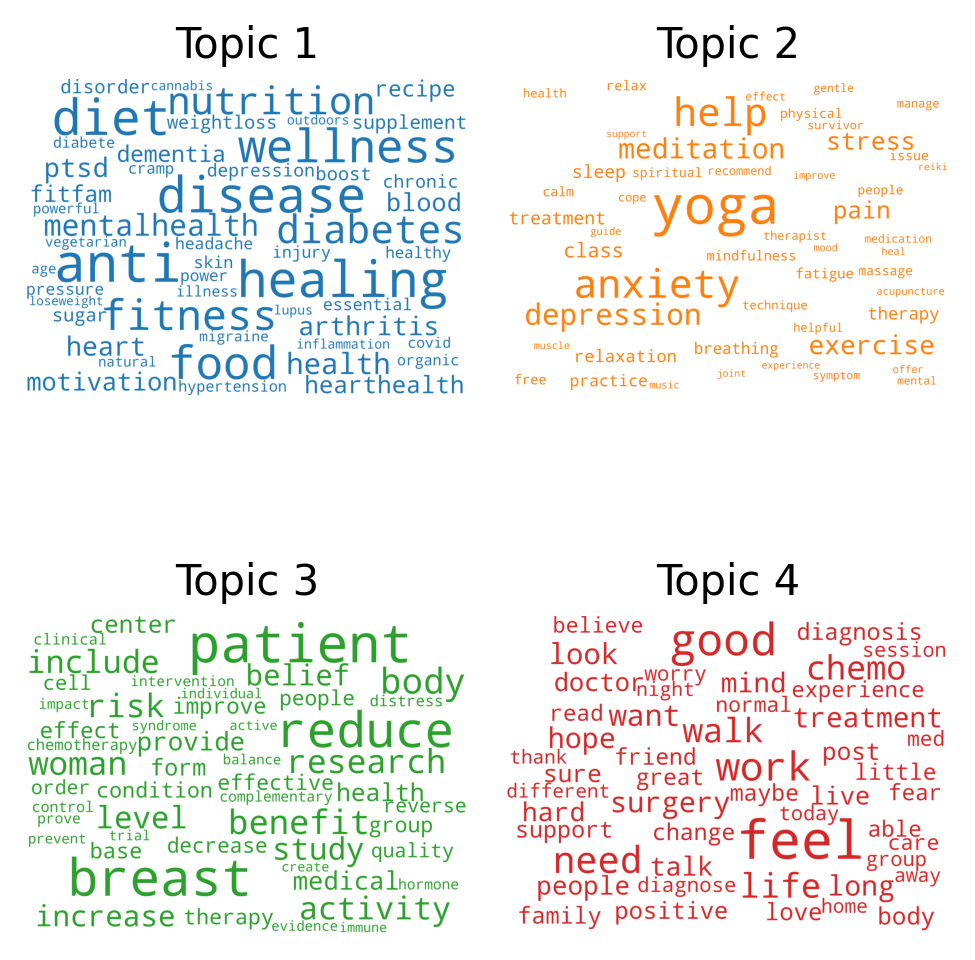


^a^ Wordclouds were generated from the results of topic modelling of texts related to each common cancer-related symptoms using unsupervised or semi-supervised LDA models. Two to twelve wordclouds were generated for exploratory analysis, and the coherence scores were compared to obtain optimal results in unsupervised models. For supervised models, we used the classification recommended by the NCCIH, and grouped the keywords into natural products; mind and body practices; and other complementary health approaches, including whole-system approaches and alternative practices, before performing topic modelling and generating wordclouds. Each wordcloud shows top 50 words of a topic detected by LDA. The font size of words are proportional to their probability of selection under the topic. Selected wordclouds that correlate symptoms with TCIM modalities were shown in the figure above. Left: anemia (e.g., vitamin, supplement); Middle: anxiety/depression (e.g., yoga, meditation); Right: lymphedema (e.g., yoga, massage)
