# Supplementary figures and images for: The Use of Traditional, Complementary, and Integrative Medicine in Cancer: Data-Mining Study of 1 Million Web-Based Posts From Health Forums and Social Media Platforms
Source: J Med Internet Res. 2023 Apr 21;25:e45408. doi: 10.2196/45408 (PMC10163397; doi:10.2196/45408)

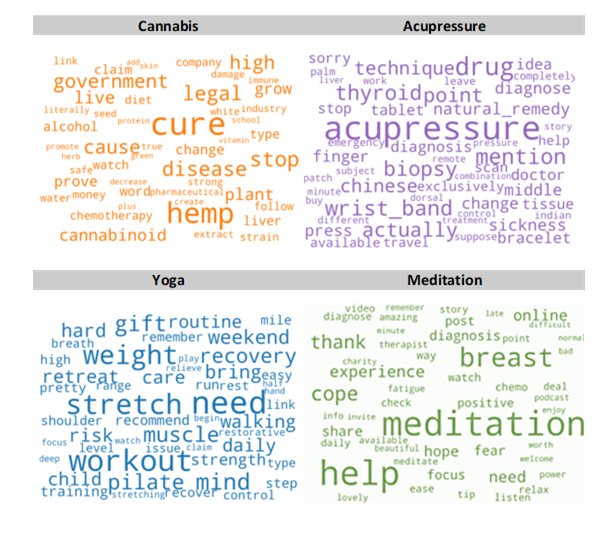

Supplement: Multimedia Appendix 2 [file jmir_v25i1e45408_app2.png]
